# Sensitivity and specificity of using trial-of-antibiotics versus sputum mycobacteriology for diagnosis of tuberculosis: protocol for a systematic literature review

**DOI:** 10.1186/s13643-018-0806-6

**Published:** 2018-09-15

**Authors:** Titus H. Divala, Katherine L. Fielding, Marriott Nliwasa, Derek J. Sloan, Ankur Gupta-Wright, Elizabeth L. Corbett

**Affiliations:** 1 London School of Hygiene & Tropical Medicine, Keppel Street, Bloomsbury, London, WC1E 7HT UK; 2 Helse Nord Tuberculosis Initiative, Department of Microbiology, University of Malawi College of Medicine, Blantyre, Malawi; 3 School of Medicine, University of St Andrews, St Andrews, UK; 4 Malawi, Liverpool Wellcome Trust Clinical Research Programme, College of Medicine, University of Malawi, Blantyre, Malawi

## Abstract

**Background:**

Suboptimal diagnostics for pulmonary tuberculosis (PTB) drives use of ‘trial-of-antibiotics (non-tuberculosis)’ in an attempt to distinguish PTB patients from those with bacterial lower respiratory tract infection (LRTI). The underlying assumption—that patients with LRTI will report ‘response’ to broad-spectrum antibiotics, while those with PTB will not—has minimal evidence base for such a widely used intervention. Numerous potential causes of misclassification include bacterial super-infection of active PTB, placebo effect, and antimicrobial resistance (AMR). The main aim of this systematic review is to collate available evidence on the performance of trial-of-antibiotics as a diagnostic test and to explore the timing, interpretation, and decision-making process.

**Methods:**

We will search MEDLINE, Embase, and Global Health using the Ovid platform for published studies that recruited adults being investigated for PTB, performed trial-of-antibiotics accompanied by mycobacteriological investigations, and reported both diagnostic test outcomes at the individual level. Following article selection, two authors will independently review titles and abstracts against eligibility criteria then perform full-text screening and extraction into a spreadsheet. We will conduct a risk of bias assessment at the level of the study using QUADAS-2 (University of Bristol) tool that assesses diagnostic evaluation work in four domains: (1) patient selection, (2) the index test, (3) the reference standard, and (4) patient flow and timing of tests. We will perform a narrative synthesis and, where possible, meta-analyses addressing our primary outcome. Our protocol adheres to the standards recommended by the PRISMA-P.

**Discussion:**

Pooling all available evidence on the accuracy, approach, and interpretation of results of trial-of-antibiotics in the context of PTB diagnosis will meet an urgent need, considering the widespread utilisation and potential for antimicrobial resistance. We therefore believe that our findings will have impact on policy and that they will inform the design of future detailed investigations into this important diagnostic approach.

**Systematic review registration:**

PROSPERO CRD42017083915

**Electronic supplementary material:**

The online version of this article (10.1186/s13643-018-0806-6) contains supplementary material, which is available to authorized users.

## Background

Limitations of current diagnostics remain a challenge in the fight against tuberculosis (TB), a leading cause of infectious disease mortality with 10.4 million new cases and 1.8 million deaths annually [1]. To complement the suboptimal diagnostics, standard diagnostic algorithms in resource-limited settings include a ‘trial-of-antibiotics.’ This is a course of broad-spectrum antibiotics, with negligible *Mycobacterium tuberculosis* activity, given to patients with symptoms such as cough in order to ‘rule-out’ or ‘rule in’ TB [2–4]. Patients with negative sputum mycobacteriology who respond to the antibiotic treatment are considered TB negative, while those who remain symptomatic are deemed likely to have TB and undergo further evaluations leading on to receiving TB treatment.

Approximately 26.5 million antibiotics courses are prescribed in the course of diagnosis of the 5.3 million smear negative TB registrations per annum [7]. This estimate is based on assuming an average of 5 antibiotic courses per sputum-negative treatment initiation, with 2 courses given to the patients before TB treatment [5] and the other 3 courses accounting for patients whose symptoms resolved and TB was ruled out [6]. Despite this widespread use, there has been no previous systematic review of the diagnostic performance of trial-of-antibiotics. The objective of this review is to assess existing evidence for the diagnostic sensitivity and specificity of using trial-of-antibiotics compared to sputum culture for TB diagnosis.

Other important evidence gaps on this subject include the choice of non-TB antibiotics (except for avoidance of those with known anti-TB activity), timing of the treatment, number of trials, the definition of treatment response, and the exact management after knowing the treatment outcome. Lack of consolidated evidence in these may be the source of the variations of implementation of trial-of-antibiotics across national programs. We will in this review consolidate existing evidence related to these gaps as our secondary objectives.

### Research question

Our study will address the following Population, Index test, Reference test, Outcome (PIRO) question.

### Objectives

#### Primary objective

Our primary objective is to determine the sensitivity and specificity of using a trial-of-antibiotics compared to sputum mycobacteriology for diagnosis of pulmonary TB (PTB).

#### Secondary objectives

Our secondary objectives are as follows:

- To describe the timing of prescription of the trial-of-antibiotics in TB diagnostic algorithms as reported in included articles
- To describe the type, duration, and number of prescriptions of routine oral antibiotics
- To establish how response to trial-of-antibiotics is interpreted and the decision-making process following positive or negative results

## Methods

### Eligibility criteria

We will include studies in any language published after 1993 that recruited adults being investigated for PTB and performed and reported outcomes of both trial-of-antibiotics and mycobacteriology investigations as part of their diagnostics work up. We will define mycobacteriology tests as any laboratory test that identifies evidence of MTB from a sputum sample. There is no defined reference mycobacteriology diagnostic test for MTB; each of the available tests has considerable flaws. Considering the time period of the review, we expect smear microscopy, smear microscopy using a fluorescent microscope, Cepheid GeneXpert, and mycobacterial culture. The guiding PIRO (population, index test, reference test and Outcome) framework for the research question is as presented in Table 1 below.

**Table 1:** Research question

Population	Adult patients with respiratory symptoms
Index test	Trial-of-antibiotics (any course of broad-spectrum antimicrobial given with the goal of ruling out TB in a symptomatic adult)
Reference test	Any mycobacteriology test (we expect smear microscopy, smear microscopy using a fluorescent microscope, Cepheid GeneXpert, and mycobacterial culture)
Outcome	Proportion of mycobacteriology-positive or mycobacteriology-negative participants correctly identified by trial-of-antibiotics (sensitivity and specificity)
Design	Cross-sectional, cohort, and randomised controlled studies

### Information sources and search strategy

We will search for studies meeting the eligibility criteria in MEDLINE, Embase, and Global Health using the Ovid platform. We will use the search strategy presented in Table 2 below to retrieve studies from the databases. We have chosen to include studies published after 1993 when the World Health Organization declared tuberculosis as a ‘global emergency’ greatly increasing funding and international commitment to tuberculosis research, management, and control efforts.

**Table 2:** Search strategy for MEDLINE using Ovid platform

Search in Ovid MEDLINE	
Search line	Search terms
Part 1	Defining study population
1.	exp Tuberculosis/
2.	tuberculosis.mp.
3.	(suspect* adj3 (TB or Tuberculosis)).mp.
4.	(presumpt* adj3 (TB or Tuberculosis)).mp.
5.	(probabl* adj3 (TB or Tuberculosis)).mp.
6.	exp Cough/
7.	tb.mp.
8.	(suspect* adj3 (TB or Tuberculosis)).mp.
9.	or/1-8
Part 2	Defining study intervention
1.	(Antibiotic* adj3 trial).mp.
2.	antibiotic*.mp.
3.	Anti-Bacterial Agents/
4.	(oral* adj3 antibiotic*).mp.
5.	(amox?cillin or erythromycin or azithromycin or doxycyclin* or Vibramycin or clavulanic acid or co-amoxiclav).mp.
6.	or/10-14
Part 3	Defining study outcome
1.	exp “Sensitivity and Specificity”/
2.	sensitivity.mp.
3.	specificity.mp.
4.	accuracy.mp.
5.	exp “Predictive Value of Tests”/
6.	((positive or negative) adj2 predictive value).mp.
7.	(ppv or npv).mp.
8.	or/16-22
Part 4	Subject combinations
1.	9 and 15 (*population and intervention*)
2.	23 and 24 (*Population and intervention and outcome*)
Part 5	Applying pre-defined limits
1.	limit 25 to yr=“1993 -Current”

In Table 2 below, we have presented our search strategy for MEDLINE, which has also been adapted for Embase and Global Health (see Additional file 1). This search strategy was reviewed by an information retrieval expert from the LSHTM library (Table 2). After completing the search in these databases, we will export results to Endnote X8 and remove all duplicates. We will also include all relevant articles identified from citations and reference lists of all included articles.

### Study selection and data extraction

Investigator TD will implement the search strategy, and then, investigators TD and MN will independently sift through titles and abstracts of the resulting papers against the eligibility criteria. TD and MN will independently assess full texts of the included papers for eligibility using the above criteria. The main reason for non-inclusion at the full-text stage will be documented. Investigator KF will resolve any disagreements in eligibility. Investigators TD and MN will then extract data from all the eligible papers into an excel spreadsheet. Should we identify multiple publications from the same study, we will report data from one.

For studies with missing or incomplete information for meta-analysis, we will contact the authors by using the contact information provided in the publications. When attempts to contact the authors have not been successful, such studies will be excluded from the meta-analysis.

### Quality assessment

We will conduct a risk of bias assessment at the level of the study using the QUADAS-2 (University of Bristol), the recommended tool for evaluating primary studies for the inclusion in systematic reviews for diagnostic accuracy. The tool, provided in Additional file 2, has four domains evaluating (1) patient selection, (2) the index test, (3) the reference standard, and (4) patient flow and timing of tests. Assessment is done with respect to risk of bias and applicability of results.

### Data analysis

We will provide a narrative synthesis of our results summarising the key findings, reporting on their consistency and quality, and identifying evidence gaps or limitations. We will perform a meta-analysis for sensitivity and specificity of trial-of-antibiotics against mycobacteriology tests for all studies providing true positives, false positives, true negatives, and false negatives. Our sensitivity-specificity joint modelling will require each study to provide data for both sensitivity and specificity.

We will utilise the MIDAS module [7] in Stata statistical software (version 15.0; Stata Corporation, College Station, TX, USA), to carry out the meta-analysis. We will also report point estimates and 95% confidence intervals, for sensitivity and specificity of trial-of-antibiotics versus mycobacteriology for each study and for pooled data, using bivariate random effects meta-analysis. We will report these results using a forest plot and plot a summary receiver operating characteristics (SROC) curve. We will examine clinical utility of trial-of-antibiotics using a Fagan plot.

### Subgroup analyses

We will perform the following subgroup analyses:

1. Geographical location. While sensitivity and specificity cannot be influenced by disease prevalence, in the case of trial-of-antibiotics, causes of symptoms and antibiotic susceptibility may vary from place to place. We will assess performance of trial-of-antibiotics versus mycobacteriology in the following regions: (1) sub-Saharan Africa, (2) Asia, and (3) South America.
2. Type of reference test. The goal of a reference standard test is to provide error-free classification of the disease outcome presence or absence. Since for TB, there is no test that truly meets this definition, the performance of trial-of-antibiotics may vary depending on the inherent properties of each reference standard. We will assess the performance of trial-of-antibiotics versus mycobacteriology in the following regions: (1) studies using microscopy-based approaches, (2) studies using MTB culture, and (3) studies using Cepheid GeneXpert.

### Assessment for heterogeneity and publication bias

We will assess the extent of heterogeneity of diagnostic specificity and sensitivity using Cochran *Q*^2^ and *I*^2^ tests. Diagnostic specificity and sensitivity forest plots and a bivariate boxplot will provide visual representation of the extent of heterogeneity.

There is limited consensus on the most appropriate approach for identifying evidence of publication bias in studies of diagnostic performance. We have decided to use the Deeks funnel plot [8], where the inverse of the square root of the effective sample size is plotted against the diagnostic odds ratio, and publication bias is deemed absent if the plot achieves a funnel shape.

## Result presentation and dissemination

For individual studies, we will present data as follows: author, year, country, whether a country has a low or high TB burden, population, sample size, design, TB reference standard, and results (sensitivity and specificity). We will present the results of our study selection using the approach prescribed by the Preferred Reporting Items for Systematic Reviews and Meta-analyses (PRISMA) [9].

We will prepare a manuscript, which we will submit for publication in a peer-reviewed journal. This work will also form part of a PhD thesis for TD, which he will submit to the London School of Hygiene & Tropical Medicine (LSHTM).

### Protocol and registration

We registered this systematic review protocol with the International Prospective Register of Systematic Reviews (PROSPERO), registration number CRD42017083915.

## Discussion

Our systematic review will be, to our knowledge, the first to pool evidence on the approach, implementation, and accuracy of using a trial-of-antibiotics for the diagnosis of tuberculosis. Trial-of-antibiotics is an integral component of diagnostic algorithms in low- and middle-income countries which, despite leading to 30 million empirical antibiotic prescriptions per annum, remains without strong evidence basis. Our findings therefore have high potential to prompt policy review as well as potentially stimulating funders and researchers to consider future studies into this component of the diagnostic algorithm.

## Additional files

Additional file 1: Search strategy for Embase and Global Health in Ovid Embase. (DOCX 18 kb)

Additional file 2: Data extraction form. (DOCX 82 kb)
