# Supplementary material for: Sensitivity and specificity of using trial-of-antibiotics versus sputum mycobacteriology for diagnosis of tuberculosis: protocol for a systematic literature review
Source: Syst Rev. 2018 Sep 15;7:141. doi: 10.1186/s13643-018-0806-6 (PMC6138901; doi:10.1186/s13643-018-0806-6)
Supplement: Supplementary file 1 — Search strategy for Embase and Global Health in Ovid Embase. (DOCX 18 kb) [file 13643_2018_806_MOESM1_ESM.docx]

# Additional file 1: Search strategy for Embase and Global Health in Ovid Embase

| **Search line** | **Search terms** |
| --- | --- |
| *Part 1 Defining study population:* | |
| 1 | exp tuberculosis/ |
| 2 | tuberculosis.mp. |
| 3 | (suspect* adj3 (TB or Tuberculosis)).mp. |
| 4 | (presumpt* adj3 (TB or Tuberculosis)).mp. |
| 5 | (probabl* adj3 (TB or Tuberculosis)).mp. |
| 6 | exp coughing/ |
| 7 | tb.mp. |
|  |  |
| 8 | 1 or 2 or 3 or 4 or 5 or 6 or 7 |
| *Part 2 Defining study intervention* | |
| 9 | (Antibiotic* adj3 trial).mp. |
| 10 | antibiotic*.mp. |
| 11 | antiinfective agent/ |
| 12 | (oral* adj3 antibiotic*).mp. |
| 13 | (amox?cillin or erythromycin or azithromycin or doxycyclin* or Vibramycin or clavulanic acid or co-amoxiclav).mp. |
|  |  |
| 14 | 9 or 10 or 11 or 12 or 13 |
| *Part 3 Defining study outcome* | |
| 15 | exp "sensitivity and specificity"/ |
| 16 | sensitivity.ti,ab. |
| 17 | specificity.mp. |
| 18 | accuracy.mp. |
| 19 | exp predictive value/ |
| 20 | ((positive or negative) adj2 predictive value).mp. |
| 21 | (ppv or npv).mp. |
|  |  |
| 22 | 15 or 16 or 17 or 18 or 19 or 20 or 21 |
| *Part 4 Subject combinations* | |
| 23 | 8 and 14 |
|  |  |
| 24 | 22 and 23 |
|  |  |
| *Part 5 Applying pre-defined limits* | |
| 25 | limit 24 to yr="1993 -Current" |

**Search in Ovid Global Health**

| **Search line** | **Search terms** |
| --- | --- |
| *Part 1 Defining study population:* | |
| 1 | exp tuberculosis/ |
| 2 | tuberculosis.mp. |
| 3 | (suspect* adj3 (TB or Tuberculosis)).mp. |
| 4 | (presumpt* adj3 (TB or Tuberculosis)).mp. |
| 5 | (probabl* adj3 (TB or Tuberculosis)).mp. |
| 6 | exp cough/ |
| 7 | tb.mp. |
|  |  |
| 8 | 1 or 2 or 3 or 4 or 5 or 6 or 7 |
| *Part 2 Defining study intervention* | |
| 9 | exp antibiotics/ |
| 10 | exp antiinfective agents/ |
| 11 | (Antibiotic* adj3 trial).mp. |
| 12 | (oral* adj3 antibiotic*).mp. |
| 13 | (amox?cillin or erythromycin or azithromycin or doxycyclin* or Vibramycin or clavulanic acid or co-amoxiclav).mp. |
|  |  |
| 14 | 9 or 10 or 11 or 12 or 13 |
| *Part 3 Defining study outcome* | |
| 15 | (sensitivity and specificity).mp. |
| 16 | (sensitivity adj5 specificity).mp. |
| 17 | sensitivity.mp. |
| 18 | specificity.mp. |
| 19 | accuracy.mp. |
| 20 | predictive value.mp. |
| 21 | ((positive or negative) adj2 predictive value).mp. |
| 22 | (ppv or npv).mp. |
|  |  |
| 23 | 15 or 16 or 17 or 18 or 19 or 20 or 21 or 22 |
| *Part 4 Subject combinations* | |
| 24 | 8 and 14 |
|  |  |
| 25 | 23 and 24 |
|  |  |
| *Part 5 Applying pre-defined limits* | |
| 26 | limit 24 to yr="1993 -Current" |
