# Supplementary material for: Sensitivity and specificity of using trial-of-antibiotics versus sputum mycobacteriology for diagnosis of tuberculosis: protocol for a systematic literature review
Source: Syst Rev. 2018 Sep 15;7:141. doi: 10.1186/s13643-018-0806-6 (PMC6138901; doi:10.1186/s13643-018-0806-6)
Supplement: Supplementary file 2 — Data extraction form. (DOCX 82 kb) [file 13643_2018_806_MOESM2_ESM.docx]

# Additional File 2: Data extraction form

**Part A: QUADAS (University of Bristol) Risk of bias assessment tool**

Use the following set of questions which use the QUADAS-2 approach to assess risk of bias of the primary study. QUADAS-2 is structured so that 4 key domains are each rated in terms of the risk of bias and the concern regarding applicability to the research question (as defined above). Each key domain has a set of signalling questions to help reach the judgments regarding bias and applicability.

| **DOMAIN** | 1. **PATIENT SELECTION** | 1. **INDEX TEST**   **(Trial-of-antibiotics**: treatment with broad spectrum antibiotics with the goal of excluding TB as a cause of respiratory symptoms**)** | 1. **REFERENCE STANDARD**   **(TB mycobacteriology** test: any sputum based microbiology test aimed at identifying evidence of TB infection**)** | 1. **FLOW AND TIMING** |
| --- | --- | --- | --- | --- |
| **Description** | Describe methods of patient selection: Describe included patients (prior testing, presentation, intended use of index test and setting): | Describe the index test and how it was conducted and interpreted: | Describe the reference standard and how it was conducted and interpreted: | Describe any patients who did not receive the index test(s) and/or reference standard or who were excluded from the 2x2 table (refer to flow diagram): Describe the time interval and any interventions between index test(s) and reference standard: |
| **Signalling questions**  **(yes/no/unclear)** | Was a consecutive or random sample of patients enrolled? | Were the index test results interpreted without knowledge of the results of the reference standard? | Is the reference standard likely to correctly classify the target condition? | Was there an appropriate interval between index test(s) and reference standard? |
|  | Was a case-control design avoided? | If a threshold was used, was it pre-specified? | Were the reference standard results interpreted without knowledge of the results of the index test? | Did all patients receive a reference standard? |
|  | Did the study avoid inappropriate exclusions? |  |  | Did all patients receive the same reference standard? |
|  |  |  |  | Were all patients included in the analysis? |
| **Risk of bias: High/low/unclear** | Could the selection of patients have introduced bias? | Could the conduct or interpretation of the index test have introduced bias? | Could the reference standard, its conduct, or its interpretation have introduced bias? | Could the patient flow have introduced bias? |
| **Concerns regarding applicability: High/low/unclear** | Are there concerns that the included patients do not match the review question? | Are there concerns that the index test, its conduct, or interpretation differ from the review question? | Are there concerns that the target condition as defined by the reference standard does not match the review question? |  |

**Part B: Exploring the study using a flow diagram**

As a way of understanding the details of each study, use the study flow diagram below. In relation to the review question and using data applicable to this review, provide numbers of participants at each stage.

For detailed comments, mark the relevant box with a number and add a corresponding details as a legend in the space below the figure.


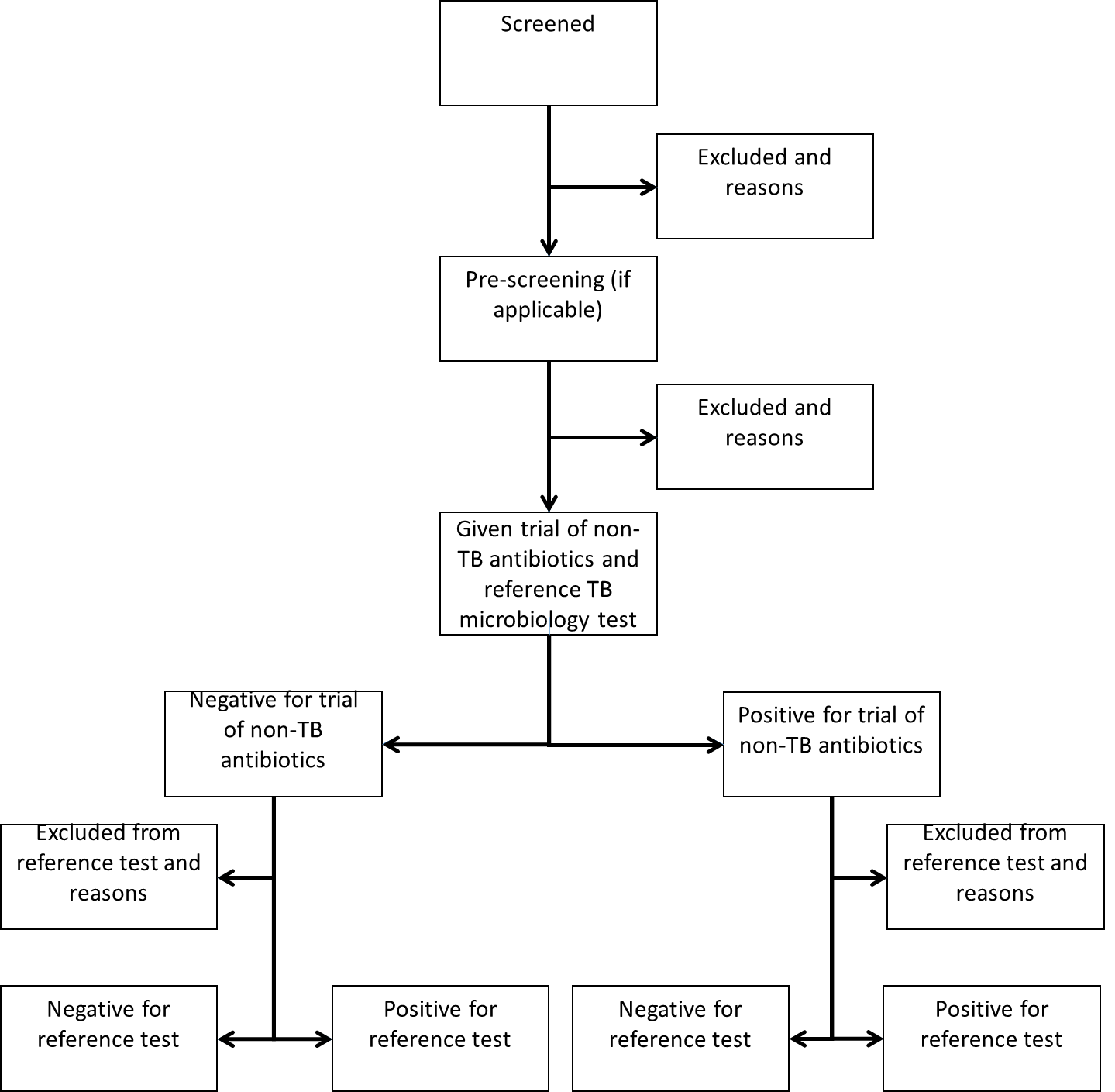


**Study design (state the design as reported by authors): .**

| **Number** | **Comment** |
| --- | --- |
|  |  |
|  |  |
|  |  |
|  |  |

**Part C: Study outcome data extraction**

Now use the following table to enter data as you extract from a primary study. The ‘response key’ section provides details of what is expected of each data field.

| **Section** | **Field** | **Response** | **Response Key** |
| --- | --- | --- | --- |
| Article identification | article_id |  |  |
|  | 1st_author |  |  |
|  | year |  |  |
|  | journal |  |  |
|  | title |  |  |
| Article eligibility | inclusion_decision |  |  |
|  | excl_reason |  |  |
| Study setting and population | country |  |  |
|  | setting |  |  |
|  | tb_suspect_defn |  |  |
|  | design |  | E.g. prospective cohort |
|  | no_participants |  | total sample size |
|  | p_hivpos |  | proportion HIV- |
|  | p_hivneg |  | proportion HIV+ |
|  | p_hivu |  | proportion HIV Unknown |
| Details of prescreening | prescreening |  | yes/no |
|  | define_prescr |  | e.g. smear negative |
| Details of test | antibiotic |  |  |
|  | abx_duration |  |  |
|  | resolution_defn |  | description of clinical resolution |
|  | mgt_nonimprove |  | What plan followed after identifying that the participant did not improve? |
|  | mgt_improve |  | What plan followed after identifying that the participant improved? |
|  | other_abx_courses |  | Describe any other antibiotic courses that were given before final TB dx decision |
| Details of reference | reference |  | Name the reference standard used to compare with outcome of trial-of-antibiotics |
|  | ref_timing |  | State the time when the reference test specimen was collected in relation to prescription of trial-of-antibiotics |
| Study profile | no_screened |  |  |
|  | no_exc_prescr |  |  |
|  | no_test_eligible |  | Number of participants eligible for trial-of-antibiotics per authors' description |
|  | no_given_trial |  |  |
|  | no_exc_trial |  |  |
|  | reas_exc_trial |  |  |
|  | without_test_resulst |  |  |
|  | reason_no_test_res |  |  |
|  | no_given_ref |  |  |
|  | no_exc_ref |  |  |
|  | reas_exc_ref |  |  |
|  | without_ref_resulst |  |  |
|  | reason_no_ref_res |  |  |
|  | no_both_ref+test |  |  |
| Antibiotic use prior to study enrollment | Prior antibioticS |  | Antibiotic use prior to study antibiotic |
|  | Proportion prior antibiotics |  | Proportion reporting taking prior to study |
|  |  |  |  |
| **Primary outcome data** |  |  |  |
| **study 2x2 table** | no_+ref+trial |  | Number positive for both reference and trial-of-antibiotics |
|  | no_+ref-trial |  | Number positive for reference but negative for trial-of-antibiotics |
|  | no_-ref+trial |  | Number negative for reference but positive for trial-of-antibiotics |
|  | no_-ref-trial |  | Number negative for both reference and trial-of-antibiotics |
